# A stony coral cell atlas illuminates the molecular and cellular basis of coral symbiosis, calcification, and immunity

**DOI:** 10.1016/j.cell.2021.04.005

**Published:** 2021-05-27

**Authors:** Shani Levy, Anamaria Elek, Xavier Grau-Bové, Simón Menéndez-Bravo, Marta Iglesias, Amos Tanay, Tali Mass, Arnau Sebé-Pedrós

**Affiliations:** 1 Department of Marine Biology, The Leon H. Charney School of Marine Sciences, University of Haifa, Mt. Carmel, Haifa 3498838, Israel; 2 Morris Kahn Marine Research Station, The Leon H. Charney School of Marine Sciences, University of Haifa, Sdot Yam, Israel; 3 Centre for Genomic Regulation (CRG), Barcelona Institute of Science and Technology (BIST), Barcelona, Spain; 4 Universitat Pompeu Fabra (UPF), Barcelona, Spain; 5 Department of Computer Science and Applied Mathematics and Department of Biological Regulation, Weizmann Institute of Science, 76100 Rehovot, Israel

**Keywords:** coral reefs, evolution, cell type diversity, Cnidaria, scRNA-seq, dinoflagellates, biomineralization, invertebrate immunity

## Abstract

Stony corals are colonial cnidarians that sustain the most biodiverse marine ecosystems on Earth: coral reefs. Despite their ecological importance, little is known about the cell types and molecular pathways that underpin the biology of reef-building corals. Using single-cell RNA sequencing, we define over 40 cell types across the life cycle of *Stylophora pistillata*. We discover specialized immune cells, and we uncover the developmental gene expression dynamics of calcium-carbonate skeleton formation. By simultaneously measuring the transcriptomes of coral cells and the algae within them, we characterize the metabolic programs involved in symbiosis in both partners. We also trace the evolution of these coral cell specializations by phylogenetic integration of multiple cnidarian cell type atlases. Overall, this study reveals the molecular and cellular basis of stony coral biology.

## Highlights

• Whole-organism single-cell analysis of the stony coral *Stylophora pistillata*
• Skeleton formation and symbiosis-associated gene expression dynamics
• Identification of immune cell programs in *Stylophora pistillata*
• Conserved broad cell type identities across cnidarian lineages

## Graphical abstract

**Figure:**
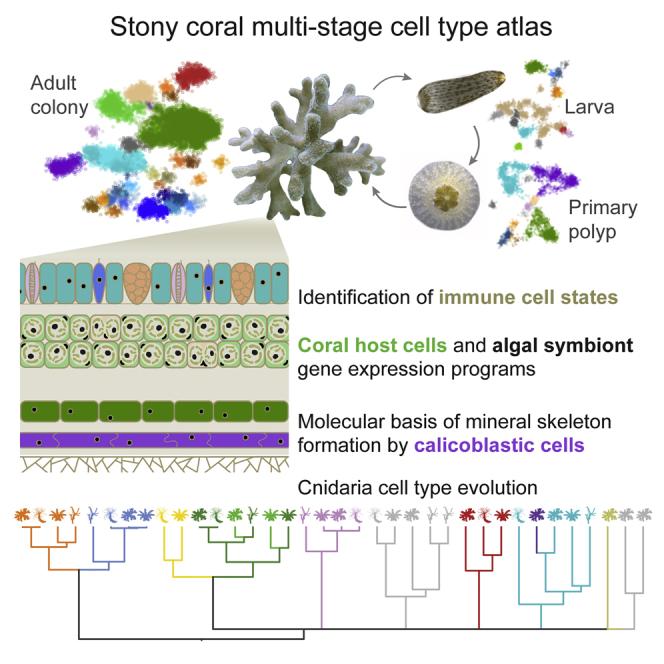

## Teaser

Over 40 cell types across the life cycle of *Stylophora pistillata*, a stony coral, are identified through the use of single-cell RNA sequencing. From this reference atlas, the molecular pathways underpinning coral reef building, specialized immune cells, and the metabolic programs involved in symbiosis between coral and algae partners are described.

## Introduction

Scleractinian corals, also known as stony corals, are the main builders of the reefs that constitute the most diverse marine ecosystems, providing home to roughly a quarter of all marine species (Reaka-Kudla, 1997). Stony corals belong to the Hexacorallia, a lineage within the Anthozoa class in the Cnidaria phylum. In addition to all of the stony corals, Hexacorallia includes sea anemones, such as the model cnidarians *Nematostella vectensis* and *Exaiptasia pallida*, and zoanthids (Baumgarten et al., 2015; Genikhovich and Technau, 2009; Technau and Steele, 2011; Zapata et al., 2015). Anthozoan life cycles involve a swimming larva that disperses, settles, and metamorphoses into a sessile polyp, which in turn develops into the adult stage. In stony corals, larval settlement is followed by rapid accretion of a protein rich skeletal organic matrix and extracellular calcium carbonate crystals (in the form of aragonite) to form a stony skeleton (Akiva et al., 2018; Vandermeulen and Watabe, 1973). Through this process of biomineralization, stony corals build the main mineral substrate of marine reefs (Drake et al., 2020; Tambutté et al., 2011).

Stony corals thrive in oligotrophic tropical and subtropical seas by forming a symbiotic consortium with photosynthetic dinoflagellate algae of the Symbiodiniaceae family (Baker, 2003; LaJeunesse et al., 2018; van Oppen and Medina, 2020). The dinoflagellate cell resides within a lysosomal-like organelle inside the host cell and transfers diverse photosynthetic products to the coral, which in turn provides the symbiont with inorganic carbon (Davy et al., 2012; Rosset et al., 2021). This photosymbiosis sustains the high-energy demands of coral growth and reproduction, including skeleton production by massive calcium carbonate deposition. In addition to diverse metabolic adaptations (Morris et al., 2019), coral immunity is considered to be a major factor for coral endosymbiosis, as well as for modulating interactions with other microbial eukaryotes and prokaryotes (Jacobovitz et al., 2019; Kwong et al., 2019).

A worldwide decline in coral reefs has been reported in the past decades (Hughes et al., 2017, 2019; Sully et al., 2019). Global changes in ocean temperature and acidification directly impact coral symbiosis and skeleton formation, causing the release of symbionts (known as coral bleaching) and reducing calcification rates, respectively (Hoegh-Guldberg et al., 2007; Putnam et al., 2017; Torda et al., 2017). The collapse in stony coral colonies has stirred investigations into the molecular basis of these unique coral specializations. To date, our understanding of the molecular biology of stony corals largely derives from genome sequencing efforts. These data have provided crucial information on coral population structure (Dixon et al., 2015; Fuller et al., 2020; Shinzato et al., 2021) and on the evolution of coral gene repertoires, including genes potentially involved in symbiosis, skeleton-formation, and immunity (Barshis et al., 2013; Bayer et al., 2012; Bhattacharya et al., 2016; Buitrago-López et al., 2020; Shinzato et al., 2011; Voolstra et al., 2017; Ying et al., 2018). However, fundamental aspects of stony coral biology are still to be clarified: the specific cellular context in which these genes are employed and the diversity of cell types encoded in scleractinian genomes.

**Figure 1:**
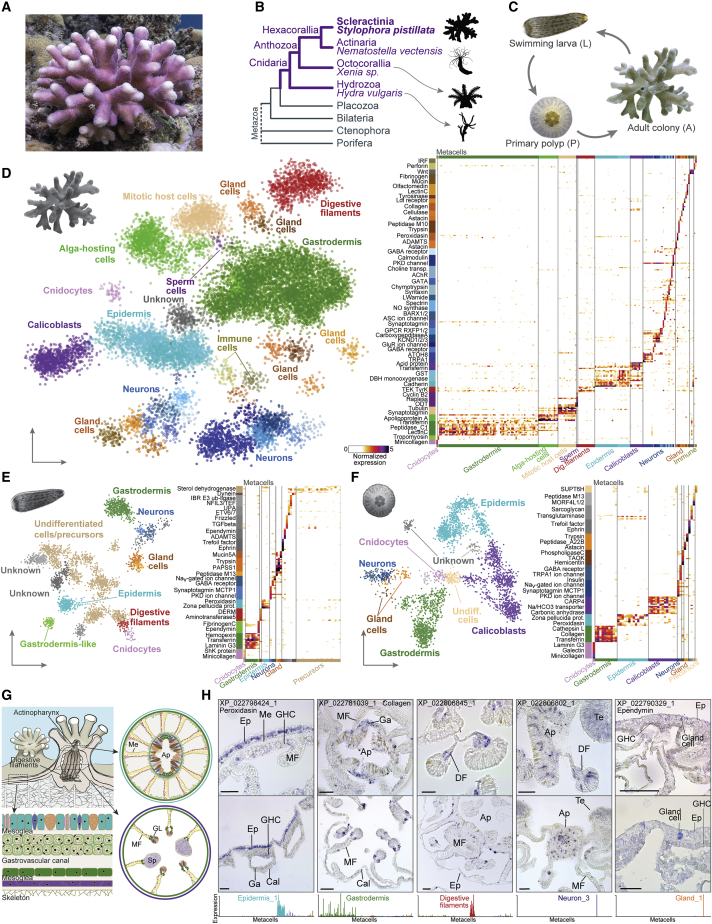
*Stylophora pistillata* multi-stage cell type atlas (A) *S. pistillata* adult colony. (B) *S. pistillata* phylogenetic position. (C) *S. pistillata* life cycle stages represented in this study. (D) 2D projection of *S. pistillata* adult single-cells (left) and normalized expression of selected variable genes across adult metacells (fold-change ≥2, allowing only a maximum of 10 genes per metacell). Broad cell types are indicated in the x axis and color bar in the y axis defines cell type in which the gene is specifically expressed. (E) Same as (D) for larva. (F) Same as (D) for primary polyp. See detailed single-cell expression maps in Figure S2. (G) Schematic representation of *S. pistillata* anatomy and tissue architecture with the major sectioning planes used for ISH experiments. (H) RNA ISH on adult tissue sections showing the expression of selected marker genes for epidermis, gastrodermis, digestive filaments, neuron_3 and gland_1. Bar plots shows the expression across metacells of the selected markers (molecules/1,000 unique molecular identifiers [UMIs]). Scale bars, 50 μm. Ap, actinopharynx; Cal, calicodermis; DF, digestive filaments; Ep, epidermis; Ga, gastrodermis; GCn, gastrovascular canal; GCv, gastrovascular cavity; GHC, gastrodermal host cell; Me, mesoglea; MF, mesenterial filaments; Sk, skeleton; Ss, skeleton spine; Sp, spermary; Te, tentacle. See also Figures S1, S2, and S3 and Tables S1, S2, and S3.

To address these questions, we used single-cell transcriptomics to systematically characterize cell type gene expression programs across the life cycle of *Stylophora pistillata*, a reef-building stony coral with a broad Indo-Pacific distribution (Figures 1A and 1B). From these whole-organism single-cell RNA sequencing (scRNA-seq) profiles, we derive detailed cell type maps for *S. pistillata* adult, primary polyp, and larval stages. Together with *in situ* hybridization validations, phylogeny-based gene annotation, and cross-species comparative analyses, these data offer insights into the molecular basis and evolution of stony coral cellular specializations, including symbiosis, calcification, and immunity.

## Results

### A multi-stage cell type atlas of *S. pistillata*

**Figure S1:**
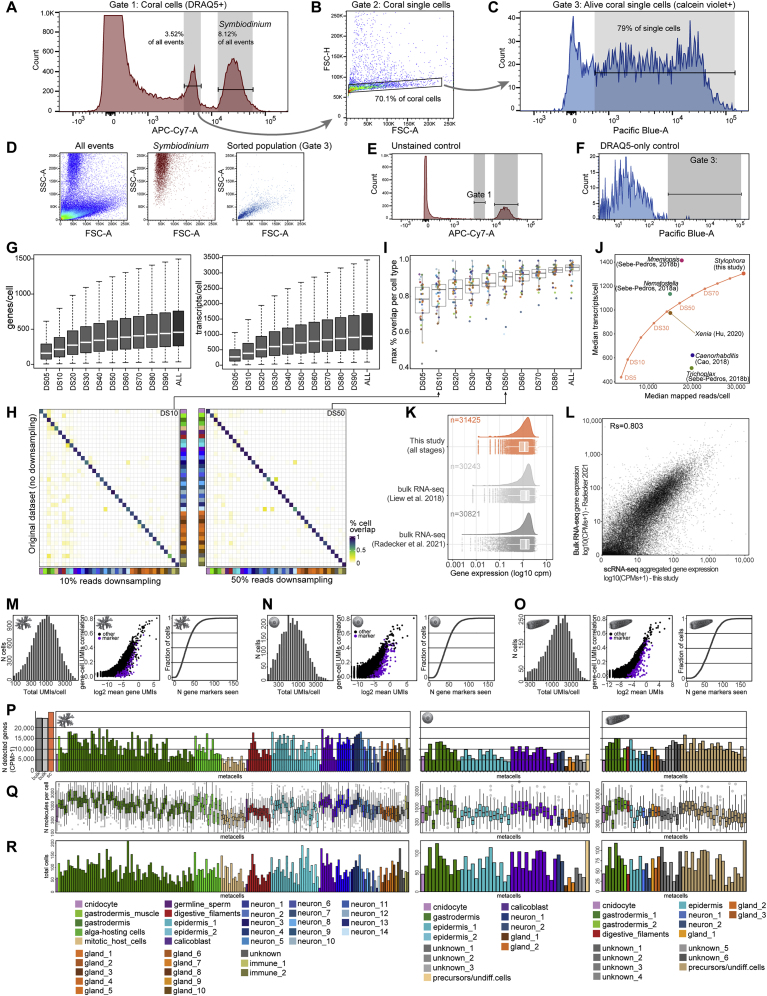
FACS-sorting strategy and *S. pistillata* scRNA-seq atlas statistics, related to Figure 1 (A) Representative flow-cytometry histogram showing DRAQ5 (dsDNA) staining of all cells and the gating strategy for selecting coral cells (Gate 1). (B) Doublet exclusion strategy (Gate 2). (C) Histogram showing calcein violet staining and gating strategy for selecting alive single cells (Gate 3). (D) FSC-A/SSC-A plots of selected populations. (E) Flow-cytometry histogram for an unstained control using the same parameters as in a. Notice the *Symbiodinium* population to the right. (F) Flow-cytometry histogram for a DRAQ5+/calcein violet- control. (G) Sequencing saturation plots. Distribution of number of genes (left) and transcripts (right) detected per single-cell in the original dataset (“ALL,” darkgrey) and downsampling the number of reads used (DS, e.g., DS50 is using 50% of the reads). (H) Cell type stability downsampling 10% (left) and 50% of the reads (right). y axis shows the original cell type groups and the y axis cell types defined in downsampled datasets. The colorscale indicate the percentage of cells within the same cell type group. (I) Summary of cell type read downsampling stability analyses. For each downsampled dataset, the percentage of cells from the original cell type definitions (colors) clustered together in the downsampled dataset is indicated (this would be equivalent to taking the diagonal values in panel I). (J) Comparison of median transcripts detected per cell (y axis) and median reads mapped per cell (x axis) in our dataset (including downsampled datasets). For reference, the values in recent whole-organism single-cell atlases are indicated. (K) Gene expression distributions in (aggregated) scRNA-seq dataset (orange) and in two bulk RNA-seq studies. (L) Comparison of scRNA-seq aggregated gene expression levels and bulk RNA-seq expression levels. Spearman correlation coefficient is indicated. (M) Left, distribution of total RNA molecules per cell in each life stage. Middle, relationship between gene total expression (x axis) and the correlation between gene expression and total RNA molecules per cell (y axis). Marker genes selected for cell clustering are shown in purple. Right, cumulative distribution of number of marker genes detected per single cell. (N) Same as M for primary polyp stage (O) Same as M for larva. (P) Number of genes detected in each metacell (CPM > 1). For comparison, the total number of genes in our scRNA-seq (light orange) and in two S. pistillata bulk RNA-seq studies (dark gray, light gray) are indicated. (Q) Total number of RNA molecules per metacell. (R) Number of cells per metacell.

To study *S. pistillata* cell type diversity, we sampled single cells from three life stages: adult stony colonies, primary polyps that are starting to produce skeleton, and non-calcifying free-swimming larvae (Figure 1C), all collected from the Gulf of Eilat/Aqaba in the Red Sea. After mechanical dissociation, we fluorescence-activated cell sorting (FACS)-sorted single cells into 384-well plates and prepared libraries using the MARS-seq protocol (Keren-Shaul et al., 2019; Figures S1A–S1F). We sequenced scRNA-seq libraries to a median of 34,846 reads/cell, approaching saturation as suggested by read downsampling analysis (Figures S1G–S1I) and reaching transcript detection levels per cell similar to other whole-organism cell type atlases (Cao et al., 2017; Hu et al., 2020; Sebé-Pedrós et al., 2018a, 2018b; Figure S1J). Globally, we detected in our scRNA-seq dataset a similar number of genes (31,425) to those seen in *S. pistillata* bulk RNA-seq studies (30,821) (Liew et al., 2018; Rädecker et al., 2021; Figure S1K), and the aggregated scRNA-seq gene expression levels correlated well with levels measured bulk RNA-seq analyses (Rs = 0.803) (Figure S1L).

**Figure S2:**
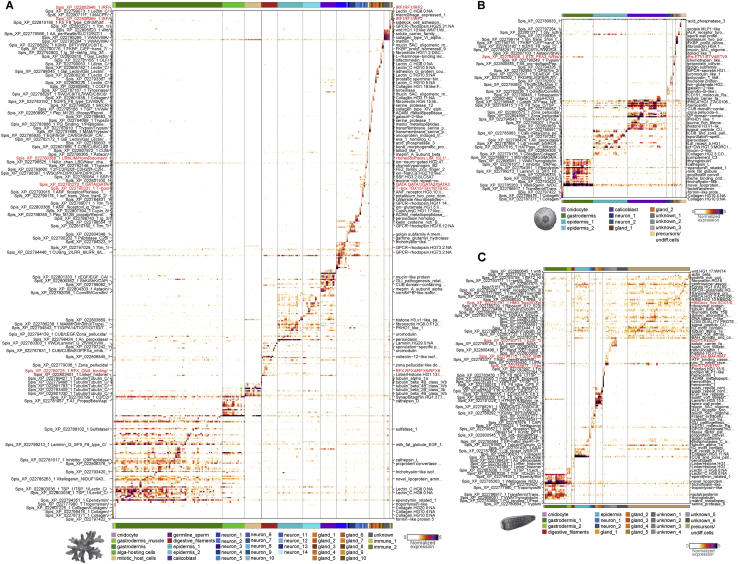
*S. pistillata* single-cell gene expression maps, related to Figures 1 and 2 (A) Expression of 428 variable genes (rows) across 16,080 adult colony single cells sorted by metacell and cell type association. (B) Expression of 158 variable genes (rows) across 3,140 primary polyp single cells sorted by metacell and cell type association. (C) Expression of 181 variable genes (rows) across 3,571 larval single cells sorted by metacell and cell type association. In all cases, top10 genes (FC > 2) per metacell are included, showing gene functional annotation when available. Transcription factors are highlighted in red.

**Figure S3:**
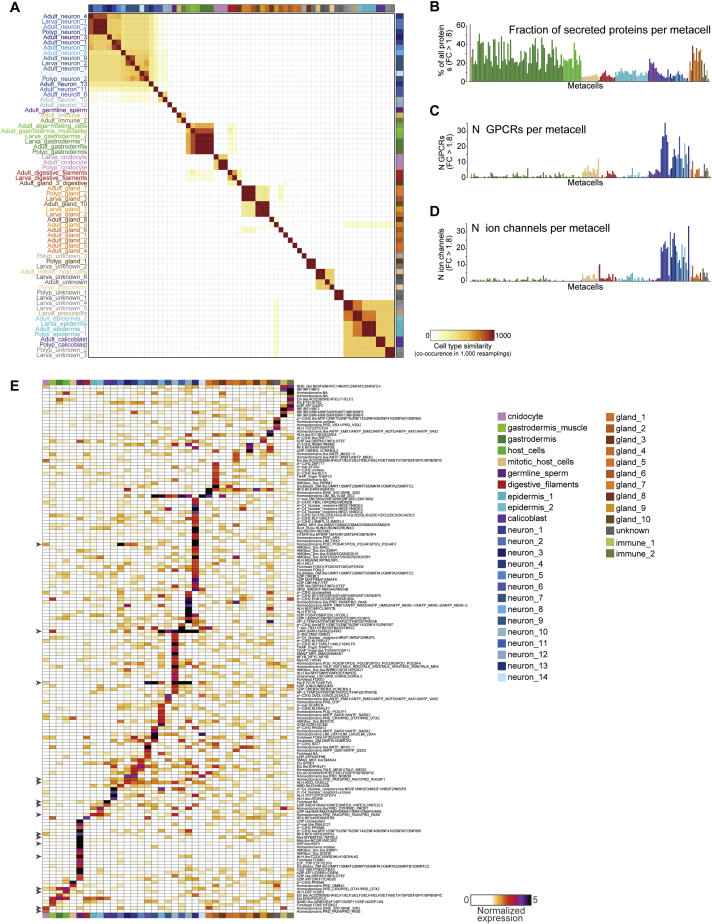
*S. pistillata* cell type similarities and adult cell atlas functional enrichments, related to Figures 1 and 2 (A) Co-occurrence matrix used to build the cell type tree in Figure 2. Cell type similarity is indicated as the number of times two cell types clustered together in 1,000 iterations with 75% downsampling of genes (see STAR Methods). (B) Percentage of predicted secreted proteins (containing a signal peptide and no transmembrane domains) among genes expressed in each metacell (fold-change > 1.8). (C) Total number of rhodopsin-type GPCRs (based on Pfam 7tm_1 domain counts) genes expressed in each metacell (fold-change > 1.8). (D) Same as (A) for ion channels (based on Pfam domain counts, see Table S2). Notice that candidate neuronal metacells contain high numbers of expressed rhodopsin-type GPCRs and voltage-gated ion channels. (E) Transcription factor expression profile across *S. pistillata* adult cell types. On the right side, the structural class and the phylogeny-based family classification for each TF is indicated (see STAR Methods). Arrows indicate TFs discussed in the main text.

After quality filtering, we retained 16,080 adult cells, 3,140 polyp cells, and 3,571 larval cells for analysis, with a median of 937, 723, and 1,373 transcripts/cell, respectively (Figures S1M–S1R). We then applied the Metacell algorithm (Baran et al., 2019; Sebé-Pedrós et al., 2018a) to group the cells into transcriptionally coherent clusters (metacells) (Figures 1D–1F, S1, and S2), which we used as the fundamental data-rich units for our downstream analyses. In each of these metacells, we detected between 5,000 and 15,000 different genes (Figure S1P). We further grouped these metacells into both specific (e.g., sperm cells) and broad cell types (e.g., neuron) based on shared co-expression of hundreds of effector genes and 208 variable transcription factors (TFs) (Figures S2 and S3). In total, we defined 37 transcriptionally distinct cell types in adult corals, 13 in primary polyps, and 17 in swimming larvae, the latter containing a large fraction of undifferentiated cells.

To confirm the expression pattern of marker genes predicted by our cell atlas model, we used *in situ* hybridization (ISH) (Traylor-Knowles, 2018; Figures 1G and 1H). We validated that (1) the epidermal gene *Peroxidasin* is expressed in cells lining the external surfaces of the adult corals; (2) a gastrodermal collagen is observed in specific cells at the edge of mesenterial lobes in the gastric cavity, as well as in cells surrounding the actinopharynx; (3) a scleractinian-specific uncharacterized protein (XP_022806845_1) is present in digestive filament cells found along the thin mesenterial filaments; (4) a collagen-like gene is found in specific neurons along the actinopharynx and in the lobes at the edge of the mesenterial filaments; and (5) a secreted ependymin domain-containing protein is expressed in large goblet-shaped gland cells scattered along the epidermis cells, facing the external environment.

**Figure 2:**
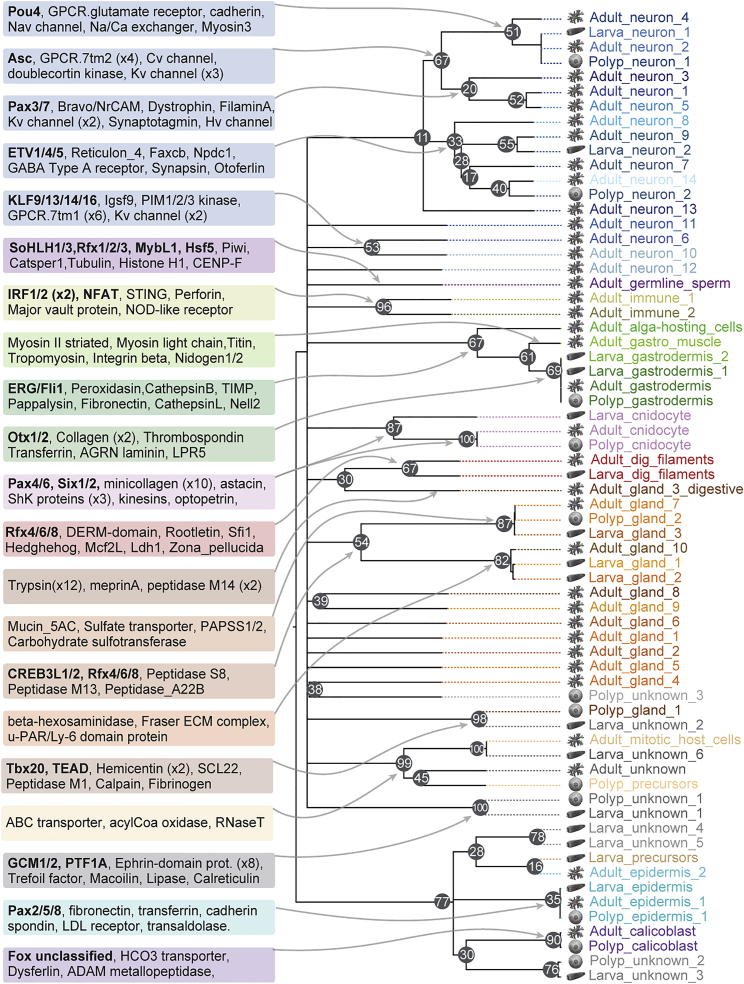
Cross-stages cell type hierarchical tree Based on hierarchical clustering of 5,881 variably expressed genes (max expression fold-change [FC] in any cell type >1.8) across all three life stages. Node values indicate percent jackknife support (with 75% downsampling). Selected genes supporting each node (FC ingroup cell types >1.7; FC outgroup <1.8, leakiness 10%) are indicated, with TFs in bold. See also Figures S2 and S3 and Table S2.

We then compared the adult, primary polyp, and larval cell type repertoires (Figures 2 and S3A). This clustering analysis identified shared cell types across the three life stages, including (1) epidermal cells, defined by *Pax2/5/8* TF, cadherins, lectins, and multiple extracellular matrix (ECM) proteins; (2) cnidocytes, expressing *Pax4/6* and *Six1/2* TFs together with dozens of secreted proteins like minicollagens and ShK toxins (Figure S3B); (3) gastrodermal cells that share expression of *Otx1/2* TF, as well as ECM and adhesion proteins; and (4) two different gland cell types and two neuronal cell types found in all three stages (Figure 2).

In addition to shared types, we also identified stage-specific cells. For instance, adult colonies contain a larger diversity of neuronal cell types, which share expression of the proneural TF *Achaete-scute* (*ASC*) (Figure S3E), the RNA-binding protein *CPEB2/3*, and large numbers of GPCRs and ion channels (Figures S3C and S3D). Neuronal cells in *S. pistillata* can be broadly divided into three groups, each one characterized by the specific expression of TFs (*Pax3/7*, *Pou4*, and *ETV1/4/5*) and distinct neuronal effector genes (e.g., ion channels, GPCRs, peptidases, and adhesion molecules). Further, adult colonies contain diverse specialized gland/secretory cell types that the other life stages lack, each expressing different combinations of peptidases, mucins, and other secreted proteins (Figures 2, S2, and S3B). One of this gland cell types (gland_3) shows expression of large numbers of trypsins and other proteases, suggesting a potential digestive cell identity (Figure 2). Another unique adult cell type is represented by sperm cells. These cells express TFs like *SOHLH1/2*, *Rfx1/3*, *HSF5*, and *MybL1*, typically associated with spermatogenesis and germline differentiation in other metazoans (Bolcun-Filas et al., 2011), as well as components of the sperm tail (*ODF* and *RSPH*) and genes involved in mitochondrial function, gamete membrane fusion (*hapless*), and cell division (*CKS*, *CyclinB2*, and *CENP-F*).

This *S. pistillata* cell atlas expands our understanding of stony coral cellular and molecular biology. The complete dataset can be explored in detail through an interactive database (https://sebe-lab.shinyapps.io/Stylophora_cell_atlas/). This web application supports visualization of cell type gene expression profiles within and between life stages, gene expression analysis, and both global and pairwise cross-species comparisons.

### Cnidaria cell type evolution

**Figure 3:**
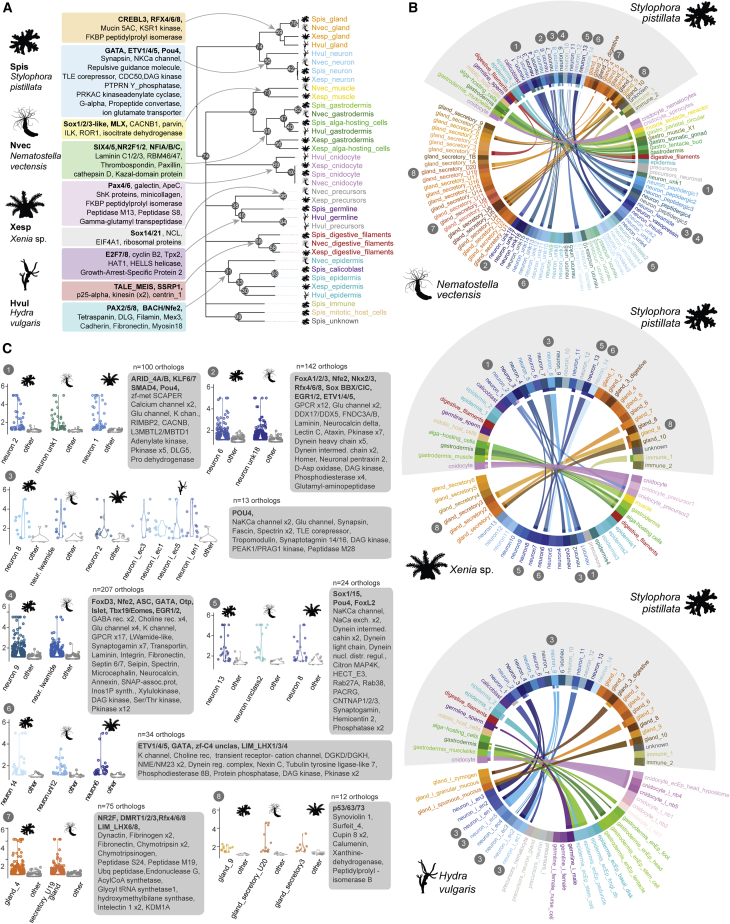
The evolution of coral cell types (A) Tree of broad cell types from four cnidarian species, based on hierarchical clustering of 1,227 variably expressed one-to-one orthologs (max expression fold-change [FC] in any cell type >1.8). Node values indicate percent jackknife support (with 75% downsampling). Selected genes supporting each node are indicated (expression fold-change ingroup cell types >1.3; FC outgroup <1.8, leakiness 10%). TFs are highlighted in bold. (B) Cross-species pairwise cell type similarities, based on Kullback-Leibler divergence (KLD). The top 2% highest values are indicated as arches connecting cell types (with a width proportional to the KLD similarity). The color annotations at the beginning at the end of each arch indicate the opposite species cell type to which it connects. (C) Examples of highly similar neuronal and gland/secretory cell types across species. Violin plots show the normalized expression of the top shared orthologous genes (FC >1.3) in each cell type, compared to the average expression of the same genes in all other cell types. The top genes in each case are indicated, with TFs highlighted in bold. Wilcoxon signed rank test adjusted p value <0.001 for all comparisons, except Hydra neuron i_ec1 and neuron i_en1 for which adjusted p value <0.01. See also Figure S4 and Table S3.

We next compared our *S. pistillata* cell atlas with available single-cell data from three other cnidarian species (Hu et al., 2020; Sebé-Pedrós et al., 2018a; Siebert et al., 2019): the solitary sea anemone *Nematostella vectensis* (Hexacorallia-Anthozoa), the soft coral *Xenia* sp. (Octocorallia-Anthozoa), and the fresh-water polyp *Hydra vulgaris* (Hydrozoa) (Figures 1B and 3). This comparative analysis offers the opportunity to study the evolution of cell type programs across three major cnidarian lineages. Although these species share a conserved polyp body-plan, they diverged from a common ancestor 500 million years ago (Park et al., 2012; Quattrini et al., 2020).

**Figure S4:**
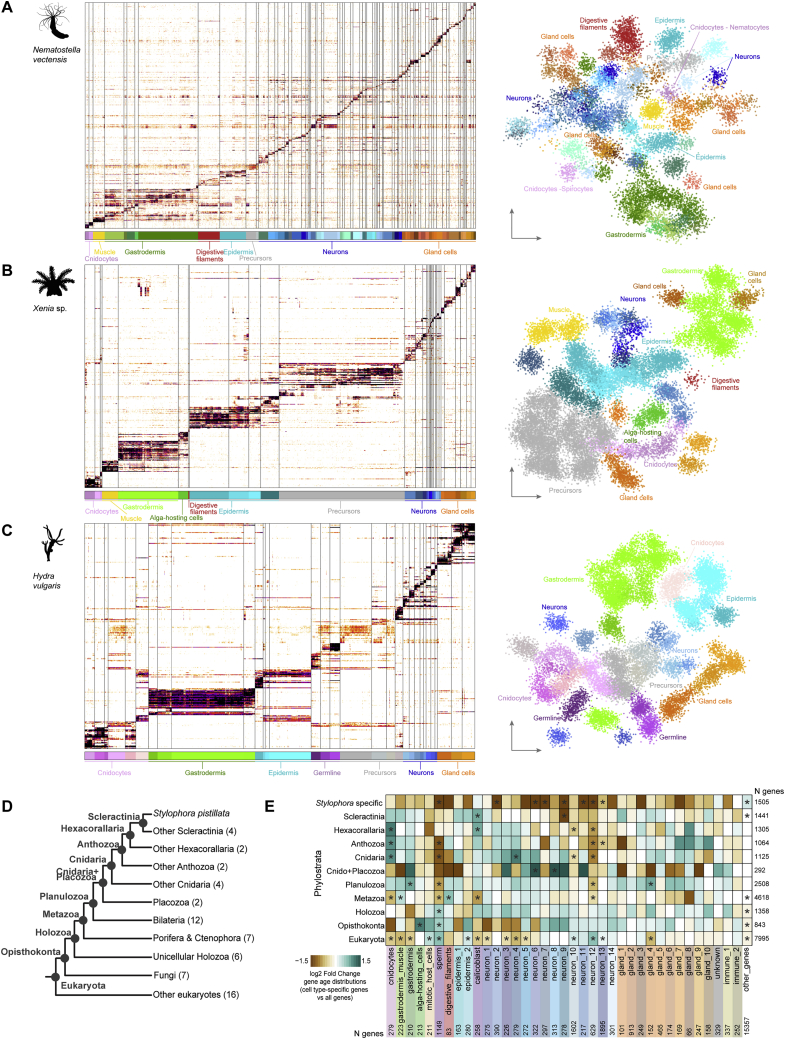
Cnidarian single-cell atlases and *S. pistillata* phylostratigraphy, related to Figure 3 (A) Expression of 627 variable genes (rows) across 13,995 *N. vectensis* single cells sorted by metacell and cell type association. (B) Expression of 391 variable genes (rows) across 24,375 *Xenia* sp. single cells sorted by metacell and cell type association. (C) Expression of 310 variable genes (rows) across 21,651 *H. vulgaris* single cells sorted by metacell and cell type association. In all cases, top 10 genes (FC > 2) per metacell are included. (D) Schematic representation of the taxon sampling used to define *S. pistillata* gene ages, indicating the number of species sampled per group. (E) Gene age enrichment/depletion in gene sets specific to each *S. pistillata* cell type (FC > 1.7). ^∗^ adjusted p value < 0.05, Fisher exact test.

To integrate cell type expression data across species, we first re-analyzed each dataset separately (Figures S4A–S4C) and integrated cell type expression matrices using 4,523 one-to-one orthologous genes found in all four species. The comparative analysis of these four cnidarian cell type atlases revealed strong transcriptional similarities at the broad cell type level, with specific co-expression of orthologous genes (Figure 3A). For example, cnidocytes of all four species shared expression of 30 orthologous genes, including the TF *Pax4/*, and anthozoan cnidocytes co-express *Pou4* and *Fos*. Moreover, neuronal cells share the expression of *synapsin*, propeptide convertases, ion channels, and multiple TFs, including *GATA*, *Pou4*, and *ETV1/4/5*. Finally, all gastrodermal/muscle cells express *Six4/5*, *NR2F1/2*, laminins, and other ECM proteins. Consistent with its more distant phylogenetic position, *Hydra* cell types show the most divergent transcriptomes compared to the three anthozoan species. For example, digestive filament cells are only found in anthozoans, where they share expression of *SSRP1* and *Meis* TFs, and flagellar apparatus gene orthologs.

Cell type level analyses revealed that *S. pistillata* shares seven direct neuronal and gland/secretory cell similarities with *N. vectensis,* five with *Xenia* sp., and only one with *H. vulgaris* (Figures 3B and 3C). For example, the three anthozoans (*S. pistillata*, *N. vectensis*, and *Xenia* sp.) share a neuronal cell type characterized by co-expression of 64 orthologs, including two voltage-gated Ca-channel orthologs together with the Ca-channel auxiliary subunit *CACNB* and the TFs *Pou4*, *ARID_4A/B*, *KLF6/7 and Smad4*. In most cases, however, we observe many-to-many neuronal similarities across species, as well as for gland cell types (Figure 3B). This pattern suggests a rapid evolutionary diversification of neurosecretory cell repertoires between major cnidarian lineages.

### Host-symbiont gene expression programs

**Figure 4:**
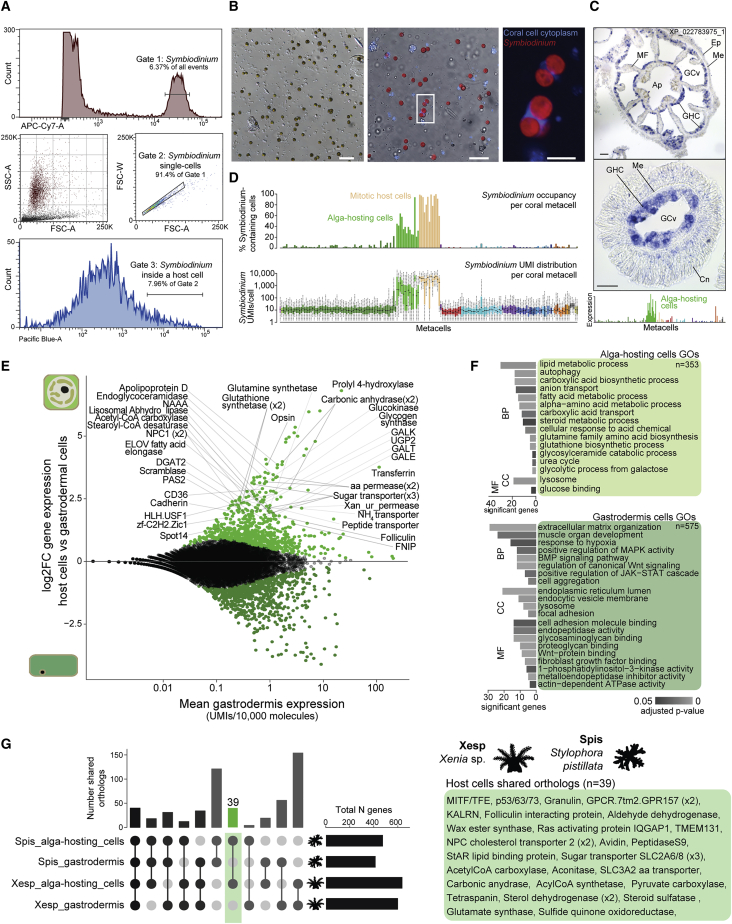
Molecular basis of stony coral symbiosis (A) FACS-sorting strategy for coral alga-hosting cells containing *Symbiodinium*. Top: *Symbiodinium*-containing cells are selected (Gate 1) based on photosynthetic pigment autofluorescence (see B). Middle: distribution of Gate 1 events among the whole cell population and doublet exclusion (Gate 2). Bottom: selection of calcein violet-positive *Symbiodinium* cells. Calcein is only incorporated by coral cells, allowing us to distinguish free/released *Symbiodinium* cells from *Symbiodinium* cells inside coral cells. (B) Bright field image of *S. pistillata* cell dissociate (left), fluorescence image showing *Symbiodinium* autofluorescence (red) and calcein violet (blue) incorporated by coral cells (right). Scale bars, 50 μm (inert, 15 μm). (C) Expression of a host cell-specific gene (XP_022783975_1) detected by using RNA ISH on adult tissue sections. Expression (blue) is found in all symbiotic algae bearing cells. Scale bars, 50 μm. Ap, actinopharynx; Cn, cnidocyte; Ep, epidermis; GCv, gastrovascular cavity; GHC, gastrodermal host cell; Me, mesoglea; MF, mesenterial filaments. (D) Top: percentage of coral cells containing a *Symbiodinium* cell within in each metacell (based on *Symbiodinium* >100 UMI counts). Bottom: *Symbiodinium* UMI count per cell in each coral metacell. (E) Differential gene expression between alga-hosting cells and gastrodermal cells. Significant genes are shown in green (adjusted p value <1e−5, Fisher exact test). (F) Enriched Gene Ontology categories for differentially expressed genes between alga-hosting cells and gastrodermal cells. (G) UpSet diagram showing shared orthologs between *Xenia* sp. and *S. pistillata* host and gastrodermal cells. See also Figures S5 and S6 and Table S3.

**Figure S5:**
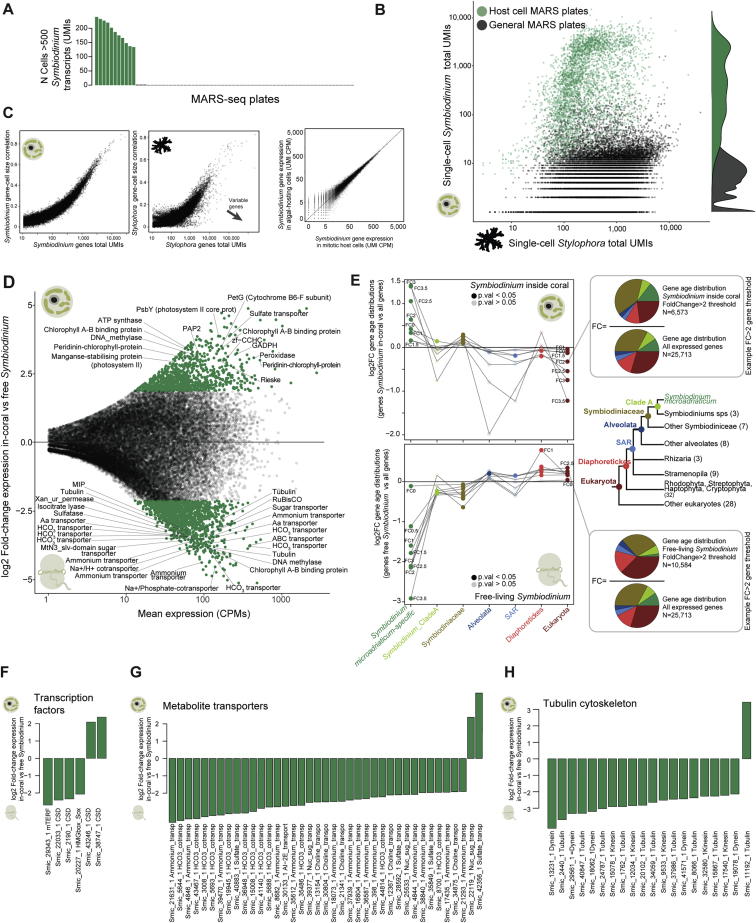
*Symbiodinium microadriaticum* single-cell analysis, related to Figure 4 (A) *in-silico* validation of FACS-sorting strategy. Barplot indicates the number of wells containing over 500 *Symbiodinium* UMIs among all 384 MARS-seq processed plates. (B) Comparison of *S. pistillata* UMIs versus *Symbiodinium* UMIs in each well. Both in A and B, dark green indicates the plates sorted with the host cell gating strategy and black indicates cells using the sorting strategy described in Figure 4A. (C) Left and middle, plots of gene-cell size correlation versus total gene expression *Symbiodinium* and *S. pistillata* cells (see STAR Methods). No variable genes are detected in *Symbiodinium* using these criteria for marker gene selection, indicating extremely low transcriptional heterogeneity among *Symbiodinium* single cells inside the coral. Right, comparison of gene expression levels between Symbiodinium cells associated to coral “alga-hosting” cell type and to “mitotic host” cell type. No significant difference in gene expression are observed. (D) Comparison of aggregated gene expression in *Symbiodinium* single cells inside the coral with bulk transcriptome data from free-living *Symbiodinium microadriaticum* (see STAR Methods). Genes with a log2 fold-change over 2 are highlighted in green and selected genes with functional annotation are indicated. (E) Phylostratigraphic analysis comparing the deviations in age distributions among differentially expressed genes between *Symbiodinium* inside the coral and free-living *Symbiodinium*. The analysis is reproduced using eight different Fold-change threshold values for defining genes overexpressed in a particular *Symbiodinium* stage. Pie charts exemplify the calculations represented in the plots for the case of a FC > 2 gene expression threshod. Dot color transparency indicates significance (Fisher exact test). (F) Normalized expression of *Symbiodinium* transcription factors. (G) Same as (F) for *Symbiodinium* metabolite transporters (identified based on Pfam domains). (H) Same as (F) for *Symbiodinium* tubulin cytoskeleton-related proteins (kinesins, dyneins and tubulins).

A critical coral cellular specialization is the establishment of a stable photosymbiosis with Symbiodiniaceae dinoflagellate algae. To gain insights into this symbiosis at single-cell resolution, we devised a FACS-sorting strategy that allowed us to sample coral cells bearing *Symbiodinium microadriaticum* dinoflagellates (Martinez et al., 2020) and to simultaneously analyze the single-cell transcriptomes of the coral cell and the dinoflagellate cell inside it (Figures 4A, 4B, S5A, and S5B). These symbionts reside inside cells found around the gastrovascular cavity and along the vascular canals in the coenenchyma, as revealed by ISH against a predicted host cell marker (XP_022783975_1) (Figure 4C).

**Figure S6:**
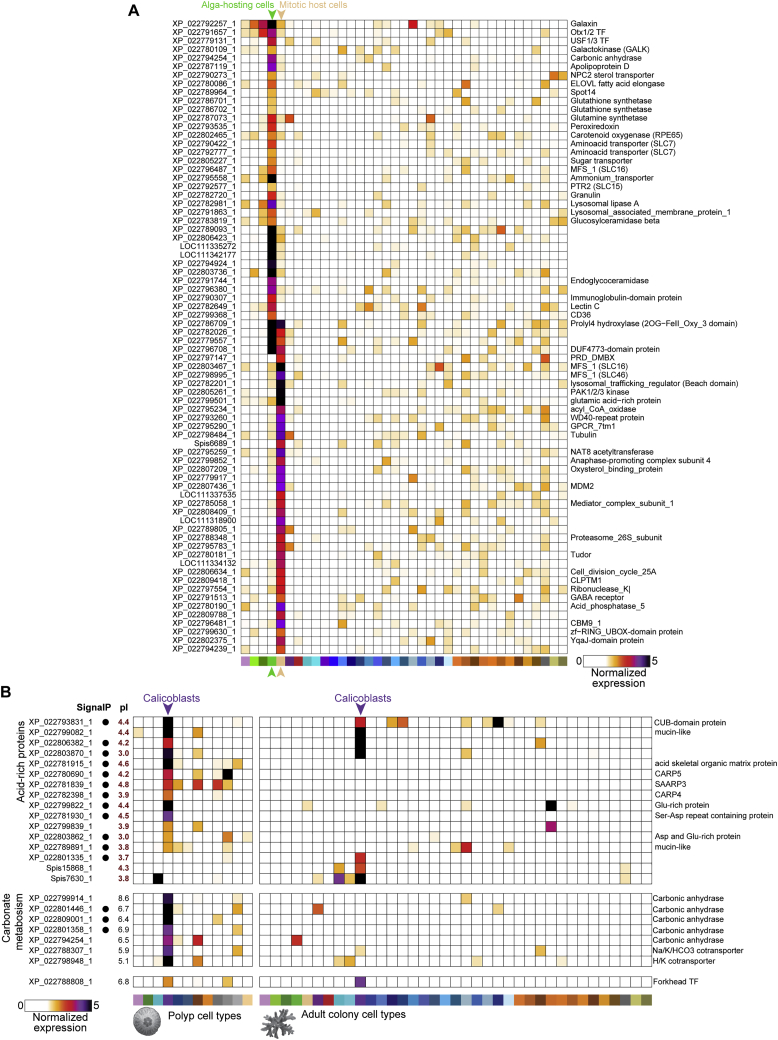
Algal-hosting cells and calicoblasts gene expression maps, related to Figures 4 and 5 (A) Gene expression for selected alga-hosting cells-related genes across *S. pistillata* adult cell types. Gene IDs and gene name annotations (when available) are indicated. (B) Gene expression for selected host cells-related genes across *S. pistillata* primary polyp and adult cell types. Gene IDs and gene name annotations (when available) are indicated, as well as the isoeletric point (pI) of the protein and the presence of a predicted signal peptide (SignalP).

First, we quantified which coral cell types contain dinoflagellate symbionts (Figure 4D): a group of gastrodermal-like cells (termed here “alga-hosting cells,” with 50% algal occupancy); and a cluster of cells that we interpret as dividing alga-hosting cells and/or germline precursors (termed here “mitotic host cells,” with 83% of algal occupancy), because they express *Nanos*, *Tudor*, multiple tubulins, and genes involved in cell cycle such as *DDB1*, *CDC25A*, and *RAE1* (Figure S6A). This group of cells contains the highest *Symbiodinium* load of all coral cells (median >5,000 molecules/cell) (Figure 4D), suggesting the presence of multiple *Symbiodinium* cells within them. Globally, *Symbiodinium* transcripts represented 18% of all sampled transcripts in our scRNA-seq dataset, which is similar to the numbers reported in bulk RNA-seq studies (Barshis et al., 2013; Mohamed et al., 2019), and when looking at individual alga-containing cells, *Symbiodinium* transcripts are between three and five times more abundant than *S. pistillata* transcripts (Figure S5B).

Next, we analyzed the expression signatures of alga-hosting cells in comparison to the closely related non-symbiotic gastrodermal cells (Figures 4E, 4F, and S6A). We identified 353 host cell-specific genes enriched in multiple metabolic functions. For example, alga-hosting cells express all the enzymes of the galactose-catabolism Leloir pathway (*GALK*, *UGP2*, *GALT*, and *GALE*), suggesting that galactose is a major carbohydrate resource obtained from the symbiont. We also found overexpression of genes involved in lipid metabolism. These include lipid transporters (*ApoD* and *NPC1*) (Hambleton et al., 2019), fatty acid elongating enzymes (*ELOV*, *PAS2*, and *DGAT2*), and transcriptional regulators typically expressed in lipogenic tissues, including *USF1* TF and *Spot14* (a regulator of acetyl-CoA carboxylase, which is also overexpressed in these cells). Alga-hosting cells express genes encoding key enzymes for glutathione production involved in the protection against oxidative stress caused by photosynthesis. They are also enriched in genes involved in *Symbiodinium* nutrient supply, such as carbonic anhydrases that ensure CO_2_ availability (Bertucci et al., 2013) and ammonium transporters (Grover et al., 2003). Alga-hosting cells also overexpress several amino acid and peptide transporters, as well as the folliculin complex (*folliculin* and *FNIP*) involved in sensing amino acid availability in the lysosomal membrane. Last, alga-hosting cells specifically express an opsin homolog, suggesting non-visual light-sensitivity and a possible role for this opsin in coordinating metabolic responses to symbiont photosynthesis (Bertucci et al., 2015; Picciani et al., 2018).

Our single-cell sampling strategy also allowed us to examine gene expression in *Symbiodinium* within the coral alga-hosting cells. The analysis of these symbiont single-cell transcriptomes revealed low-heterogeneity (Figure S5C), indicating similar cell states of the symbiont within the coral. Next, we compared the aggregated expression of *Symbiodinium* inside corals with existing transcriptome data from a non-symbiotic *Symbiodinium* culture (Liew et al., 2017; Figure S5D). Globally, we detected 29,016 *Symbiodinium* genes in our scRNA-seq dataset, as compared with 30,500 in bulk-RNA-seq. This difference could be explained by limited sensitivity of scRNA-seq and/or by biological differences between *Symbiodinium* inside coral cells versus free-living *Symbiodinium*. When comparing the expression of genes detected in both datasets, we observed differential expression of CSD and HMG-box TFs between the two stages (Figure S5F), whereas free-living cells show high expression of diverse molecular transporters and tubulin-cytoskeleton-related genes that are generally less expressed in *Symbiodinium* inside corals (Figures S5G and S5H). Interestingly, many of these transporters are expressed by coral alga-hosting cells (e.g., ammonium and ion transporters) (Figures 4E and S6A), indicating metabolic complementation between the coral and the symbiont. We then examined the age distribution of differentially expressed *Symbiodinium* genes. This phylostratigraphic analysis suggested that the expression of recent (evolutionarily young) genes is enriched in *Symbiodinium* inside the coral (Figure S5E). We observed a similar enrichment pattern when controlling for the large number of gene duplications in *Symbiodinium* genome (Aranda et al., 2016) by removing all genes with inparalogs (data not shown). This observation indicates the possibility that many symbiosis-related genes originated within the Symbiodiniaceae lineage.

Finally, using the cnidarian comparative framework described above, we explored the evolution of *S. pistillata* host cells transcriptional program. We observed that *S. pistillata* host cells more closely resemble gastrodermal cells than symbiont-bearing host cells from the octocorallian *Xenia* sp. (the only other symbiotic cnidarian species in our analyses) (Figures 3A and 4G). This suggests that alga-hosting cells arose independently from gastrodermal-like cells in these two distantly related cnidarians. However, we found that alga-hosting cells in *S. pistillata* and *Xenia* sp. share expression of multiple genes that are not detected in their corresponding non-symbiotic gastrodermal cells, many of which are related to lipid metabolism (e.g., *StAR* and *NPC* sterol transporters and sterol dehydrogenases), as well as include orthologous amino acid and sugar transporters and two TFs (*MITF* and *p53*) (Figure 4G). This suggests a convergent mobilization of similar molecular pathways in the gastrodermal-derived host cell state.

### The molecular basis of stony coral skeleton formation

**Figure 5:**
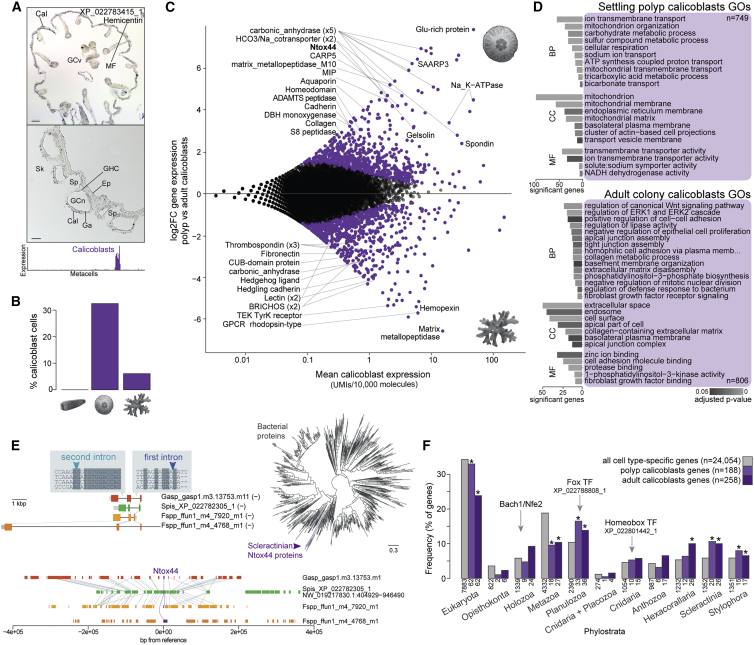
Expression dynamics of calcium carbonate skeleton formation (A) Expression of the calicoblast marker XP_022783415_1. Expression (blue) is found in the external cells surrounding the aboral region of the primary polyp (top) and in cells lining skeleton spines and gastrovascular canals, interconnecting adult colony polyps (bottom). Scale bars, 50 μm. Cal, calicodermis; Ep, epidermis; Ga, gastrodermis; GCn, gastrovascular canal; GCv, gastrovascular cavity; MF, mesenterial filaments; Sk, skeleton; Sp, skeleton spine. (B) Fraction of calicoblastic cells in each sampled life stage. (C) Differential gene expression between primary polyp calicoblasts and adult colony calicoblasts. Significant genes are shown in purple (adjusted p value <1e−5, Fisher exact test). (D) Gene Ontology analysis for differentially expressed genes between polyp and adult calicoblasts. (E) Top left: schematic representation of the intron/exon structure in the four scleractinian *Ntox44* genes. All four genes share two homologous introns, as indicated by their insertions in conserved positions in the aligned spliced transcripts. Top right: phylogenetic tree of genes carrying *Ntox44* domains in bacteria and three scleractinians (purple). Bottom: conserved synteny around the *Ntox44* locus in *S. pistillata*, *Goniastrea aspera*, and *Fungia* sp. (two paralogs). *Ntox44* is highlighted in purple. Gray lines highlight pairs of homologous genes relative to *S. pistillata* (based on diamond pairwise alignments). Synteny is conserved across all three species, except for one species-specific paralog in *Fungia* sp. (F) Gene age distribution of calicoblast-specific genes (defined as genes with an expression FC in calicoblasts >1.7) in settling polyps (light purple) and adults (dark purple) as compared to the distribution of all variable genes (gray, gene expression FC in any cell type >1.7). The inferred origin of TFs unique to calicoblasts is indicated. ^∗^Adjusted p value < 0.05, Fisher exact test. See Figure S4D for details on phylostrata definition. See also Figure S6 and Table S3.

Another unique cellular specialization in stony corals are calicoblasts, the cells involved in the production of the calcium carbonate skeletons that constitute the mineral substrate of coral reefs (Drake et al., 2020; Mass et al., 2013; Tambutté et al., 2011). We identified candidate calicoblastic cells in our single-cell dataset and validated the expression of the predicted calicoblast marker XP_022783415_1 by ISH. We detected expression of this gene in the thin external layer of the polyp, in the aboral region, and in cells lining skeleton spines and the interconnecting gastrovascular canals (Figure 5A).

Cross-stage comparisons showed that calicoblasts are absent in the swimming larvae; but very abundant in settling polyps (>30% of sampled single cells) and detected in lower frequencies in colonial adults (<7%) (Figure 5B). We also found 749 and 806 genes that are specifically expressed in calicoblasts from polyps and adults, respectively (Figures 5C and 5D). The transcriptome of settling polyp calicoblasts is enriched with genes involved in mitochondrial functions, transmembrane channels and transporters (e.g., aquaporins), ECM proteins, and remodeling peptidases. It is also enriched with genes associated to carbonate formation and transport, including five carbonic anhydrases (CO_2_ ←→ HCO_3_) and two HCO_3_/Na cotransporters (Bertucci et al., 2013; Zoccola et al., 2015); as well as enriched in acid-rich proteins often containing a secretory signal peptide (Figure S6B) and known to be highly abundant in coral skeleton proteomes (Drake et al., 2013; Mass et al., 2013; Ramos-Silva et al., 2013). In addition, we identified an homolog of a bacterial secreted RNase toxin (*Ntox44*) with potential antimicrobial functions (Zhang et al., 2012) that is highly expressed in primary polyp calicoblasts. Phylogenetic analysis indicates that this toxin derives from an ancient event of horizontal gene transfer in the scleractinian lineage. This is inferred by the presence of *Ntox44* homologs of bacterial origin in three different scleractinian species, all of which are intronized, contain an N-terminal secretion signal peptide, and show conserved intronic positions and syntenic genomic neighborhoods (Figure 5E). We hypothesize this *Notx44* homolog may play a role in clearing the settling substrate, however, further studies will be needed to clarify the role of this poorly known protein in *S. pistillata* and other scleractinians. Adult calicoblasts mainly express cell-cell and cell-ECM adhesion genes, including lectins, thrombospondins, and nidogen. Despite these differences in effector genes, calicoblast identity is maintained by a shared Fox TF homolog (XP_022788808_1) both in polyps and adults. In contrast, in the primary polyp, calicoblasts differentially express a Homeobox TF (XP_022801442_1), whereas in adults, calicoblasts express *Bach/Nfe2* and *Pax2/5/8* TFs. The latter is also expressed in epidermal cells. These results indicate a developmental shift in calicoblast function: from energy metabolism, fast skeleton-formation, and substrate settling in primary polyps, to an epidermal-like identity dominated by cell adhesion functions in adults.

The comparative analysis of cell types across Cnidaria shows that *S. pistillata* calicoblasts are transcriptionally similar to *S. pistillata* epidermis, suggesting this is a cell type novelty within *S. pistillata* or within the scleractinian lineage (Figure 3A). To gain further insights into the evolution of the calicoblastic cell type program, we performed a phylostratigraphic analysis of genes expressed in calicoblasts both in the adult and polyp stages (Figures 5F, S4D, and S4E). This analysis revealed significant enrichment of genes originated in Hexacorallia and Scleractinia, or unique to *S. pistillata* among genes expressed both in adult and settling polyp calicoblasts. In contrast, the three calicoblast-specific TFs have an earlier evolutionary origin. These results suggest that calicoblasts originated from epidermal cells during scleractinian evolution. In this process, ancestral TFs—not linked with epidermal functions—recruited novel genes, specific to Scleractinia or *S. pistillata*, to form a new cell type.

### Coral immune cell types

**Figure 6:**
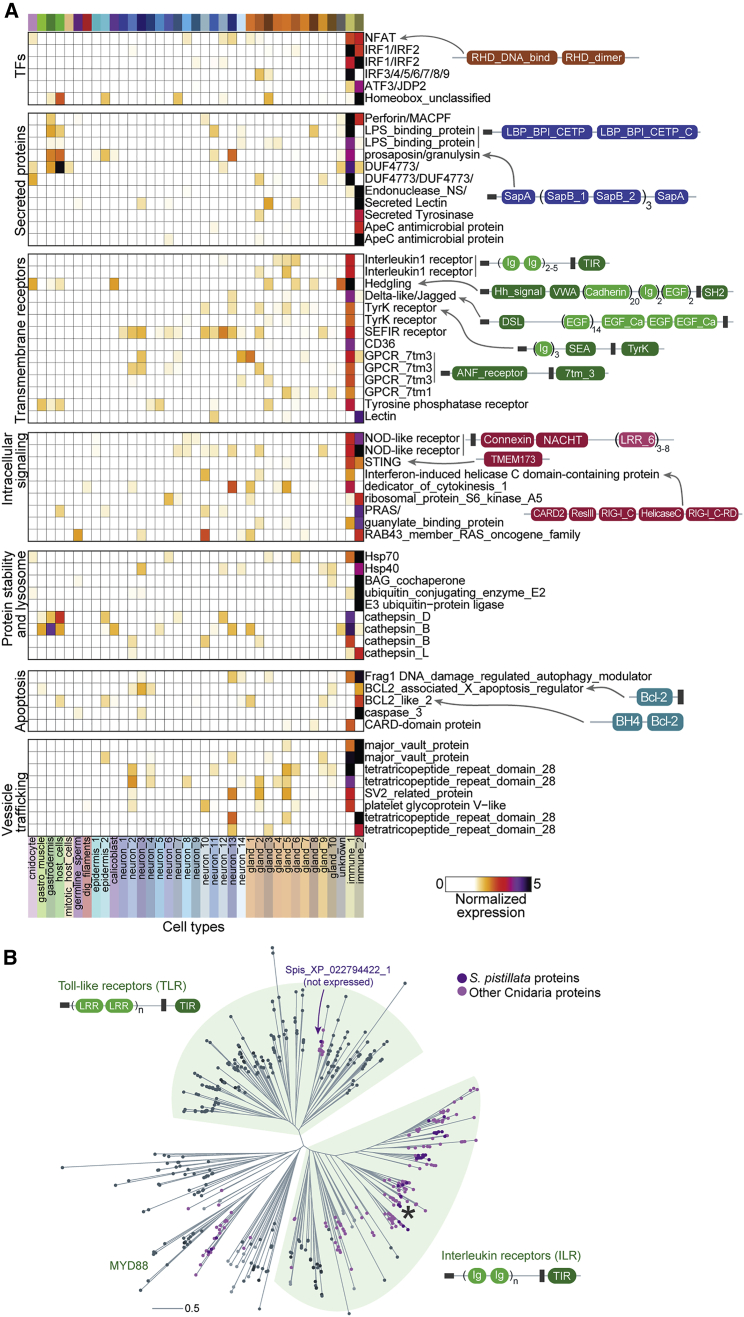
Stony coral immune cell type programs (A) Gene expression for selected immune-related genes across *S. pistillata* cell types. Protein domain architectures are indicated, including signal peptide and transmembrane domains in gray. (B) Phylogenetic tree of TLR/ILR genes in animals, highlighting *S. pistillata* genes (dark purple) and other cnidarian homologs (light purple), and the archetypical protein domain architectures of the TLR and ILR families. All gene trees have been obtained from maximum likelihood phylogenetic analyses with *IQ-TREE* (see STAR Methods).

Coral genomes encode multiple genes potentially involved in innate immune responses (Miller et al., 2007; van Oppen and Medina, 2020; Palmer and Traylor-Knowles, 2012; Walters et al., 2020). It has been hypothesized that innate immunity is an important component of symbiotic homeostasis and coral resilience to environmental stressors (Palmer and Traylor-Knowles, 2012), but to date, no specialized immune cells have been reported in corals or any other cnidarians. Our single-cell analysis identified two distinct cell types with molecular signatures indicative of immune function (Figure 6A). Both coral immune cell types express *NFAT* (a TF expressed in most immune cells) (Macian, 2005), two *Interferon Regulatory Factor* (*IRF1/2*), and other immune genes (like *STING/TMEM173* and two NOD-like receptors). Beyond these similarities, the first immune cell type specifically expresses the *IRF3/9* TF, two transmembrane interleukin-1 receptor orthologs (Figure 6B), and numerous secreted proteins (*Perforin/MACPF*, prosaposins, and LPS binding proteins). In contrast, cells in the second immune population specifically express *Atf3/JDP2* and a cnidarian-specific Homeobox TF. Moreover, they are enriched in transcripts for multiple secreted proteins: a broad spectrum endonuclease, antimicrobial ApeC proteins, and *Tyrosinase*, which is responsible for melanization (an important process in invertebrate immune response) (Cerenius et al., 2010; Palmer et al., 2008, 2011). Finally, the second immune cell type also overexpresses genes involved in apoptosis (*Bcl2*, *PARS*, and *Frag1*), protein turnover, and inflammatory response.

Overall, these findings indicate the existence of a specialized cellular immune system in corals and uncover the gene expression programs associated with coral immune function. Importantly, the involvement of conserved TF regulators (*NFAT* and IRF orthologs) and effector genes implies the existence of an evolutionary conserved transcriptional program for immune response across animals.

## Discussion

The whole-organism multi-stage *S. pistillata* cell atlas presented here uncovers the diversity of cell types in stony corals, advancing our understanding of the molecular pathways and regulatory programs involved in reef formation. Despite their ecological importance and decades of study, stony corals are still far from becoming stable model species, in part due to the difficulty of growing and reproducing them in laboratory conditions. A recent important development has been the first proof-of-concept application of CRISPR/Cas9 for gene knockout in a stony coral (Cleves et al., 2018). This opens the window to functional studies, but the definition and prioritization of target study genes is still a major limitation (Cleves et al., 2020). Our *S. pistillata* atlas constitutes a valuable resource in this direction, providing hundreds of marker genes for dozens of specific cell types. For example, we found that alga-hosting cells express genes associated with the lysosomal compartment and also genes involved in diverse lipid metabolic processes (from transport to fatty acid elongation and also a lipid metabolism-related TF-*USF1*), in protection against oxidative stress, in galactose metabolism, and in transport of other different metabolites (sugars, amino acids, ammonium, etc.). Similarly, we could identify genes overexpressed in the calicoblasts of skeleton-producing polyps, including diverse carbonic anhydrases, which catalyze the interconversion of CO_2_ to carbonate and a proton (H^+^) necessary to biomineralization process; as well as and acid-rich proteins, which are thought to be involved in calcium carbonate precipitation (Mass et al., 2013). Finally, we identify two putative immune cell types in corals, characterized by expression of genes typically associated with immune signaling pathways (interleukin receptors, *STING*, and NOD-like receptors), antimicrobial responses (e.g., perforins, LPS binding proteins, and tyrosinase), and immune cell identity transcription factors (*NFAT* and several interferon regulatory factors).

The *S. pistillata* cell atlas also allowed us to initiate the comparative analysis of cnidarian cell types by phylogenetic integration of other cnidarian cell atlases. This multi-species single-cell comparison provides systematic evidence of the evolutionary conservation of major cnidarian cell type programs (e.g., cnidocytes, neurons, secretory/gland cells, digestive filament cells, and gastrodermal cells). Beyond that, we found evidence of deep conservation in sperm and immune cell type transcriptional programs between corals and other animal lineages, reflecting highly specialized and ancient effector gene repertoires. In contrast with these conserved expression programs, our study also traces the emergence of stony coral cell type novelties: calicoblasts and alga-hosting cells. The calicoblasts emerged from epidermal cells during scleractinian evolution and this was likely a pivotal event in the origin of coral reef ecosystems. Reconstructing the detailed molecular changes involved in the emergence of the calicoblast transcriptional program, however, will require the analysis of multiple additional cnidarian species, especially other scleractinians. In the case of *S. pistillata* alga-hosting cells, they transcriptionally resemble *S. pistillata* gastrodermal cells, and this pattern is paralleled in *Xenia* sp., a distantly related symbiotic cnidarian species. This suggests the independent evolution of dinoflagellate symbiosis in these two cnidarians, which is further supported by the apparent promiscuity of dinoflagellate symbioses across animal phyla (Melo Clavijo et al., 2018). Interestingly, some common transcriptional signatures are shared between alga-hosting cells in both cnidarian species, particularly genes related to lipid metabolism. Some of these shared genes, like NPC sterol transporters, have also been found associated to symbiosis in the anthozoan anemone *Exaiptasia pallida* (Hambleton et al., 2019). Future single-cell sampling efforts in cnidarian and non-cnidarian symbiotic species should elucidate the convergent evolution of dinoflagellate symbioses across animals.

Single-cell transcriptomics has emerged as a powerful tool to explore the diversity of cell types in non-traditional model animal species (Fincher et al., 2018; Musser et al., 2019; Sebé-Pedrós et al., 2018a, 2018b; Siebert et al., 2019; Sladitschek et al., 2020). The increase in cell maps for phylogenetically diverse species should enable the comparative study of how animal cell type programs emerged and evolve (Arendt et al., 2016). However, single-cell taxon sampling efforts are almost exclusively limited to species that can be grown in laboratory conditions. Consequently, the number of metazoan species and phyla with cell atlases available remains surprisingly small, especially if we compare it with genome data availability (Dunn and Ryan, 2015). In this context, our study shows the power and feasibility of single-cell analysis in species sampled from the wild. As exemplified here, and together with improvements in sampling strategies (García-Castro et al., 2020), we anticipate that a phylogenetically rich animal cell type tree of life should be within reach in the coming years.

Overall, the *S. pistillata* cell atlas lays the foundations for a system-level molecular understanding of reef-building stony corals. This will empower the design and interpretation of studies on how environmental stressors linked to global change alter the normal function of stony coral cells. Beyond that, our cellular roadmap should enable the development of targeted strategies to improve coral resilience to global change, ultimately impacting the reef ecosystems that depend on stony coral health.

### Limitations of study

Our study provides a reference molecular map of transcriptional cell states in *Stylophora pistillata*. There are several preliminary observations reported here that will deserve follow-up analyses to extend our understanding of stony coral cell biology. Among these, we want to highlight: (1) the involvement of Ntox44 homologs in polyp settlement, including the structure and function of this poorly characterized gene family that has been horizontally transferred from bacteria to scleractinian corals; (2) the role of the opsin gene ortholog expressed in algal-hosting cells, potentially coupling symbiont photosynthesis with host cell metabolic states; and (3) the tissue distribution and function of the immune cells identified here. Finally, we did not identify any adult progenitor or stem cell-like populations in our atlas. Future sampling efforts will be required to try to identify these cells in *S. pistillata* and other stony corals.

## STAR★Methods

### Key resources table

**Table:** 

REAGENT or RESOURCE	SOURCE	IDENTIFIER
**Antibodies**		

Anti-Digoxigenin-AP, Fab fragments	Roche	Cat#11093274910; RRID: AB_2734716

**Biological samples**		

*Stylophora pistillata* adult, swimming larva and settled polyp specimens	Collected from the wild, Gulf of Eilat, Israel.	N/A

**Critical commercial assays**		

Draq5	ThermoFisher Scientific	Cat#62251
Calcein Violet AM	ThermoFisher Scientific	Cat#C34858
Liberase TM	Roche	Cat#05401119001
MAXIscript T7 Transcription Kit	ThermoFisher	Cat#AM1312
BCIP/NBT	Roche	Cat#11681451001
Blocking reagent	Merck	Cat# 11096176001
Nucleospin RNA clean-up kit	MACHEREY-NAGEL	Cat#740903
Digoxigenin labeled UTP	Roche	Cat#3359247910

**Deposited data**		

Raw and analyzed data	This paper.	GSE166901
Gene expression and annotation tables.	This paper.	https://doi.org/10.17632/g2mysjfp52.1

**Experimental models: organisms/strains**		

*Stylophora pistillata* adult colony wild type	Collected from the wild, Gulf of Eilat, Israel.	N/A
*Stylophora pistillata* larvae wild type	Collected from the wild, Gulf of Eilat, Israel.	N/A

**Oligonucleotides**		

MARS-seq barcoded primers for mRNA capture	Keren-Shaul et al., 2019	N/A
Primers for ISH probes (see Table S3)	This paper.	N/A

**Software and algorithms**		

STAR	Dobin et al., 2013	https://github.com/alexdobin/STAR
MetaCell	Baran et al., 2019	https://github.com/tanaylab/metacell
Broccoli	Derelle et al., 2020	https://github.com/rderelle/Broccoli
HMMER3	Mistry et al., 2013	http://hmmer.org/
DIAMOND	Buchfink et al., 2015	https://github.com/bbuchfink/diamond
MCL	Enright et al., 2002	https://micans.org/mcl/
MAFFT	Katoh and Standley, 2013	https://mafft.cbrc.jp/alignment/software/
ClipKIT	Steenwyk et al., 2020	https://github.com/JLSteenwyk/ClipKIT
IQ-TREE	Nguyen et al., 2015	http://www.iqtree.org/
ETE3	Huerta-Cepas et al., 2016b	http://etetoolkit.org/
eggNOG-mapper	Huerta-Cepas et al., 2017	https://github.com/eggnogdb/eggnog-mapper
topGO	Alexa et al., 2006	https://bioconductor.org/packages/release/bioc/html/topGO.html

**Other**		

*Stylophora pistillata* genome	Voolstra et al., 2017	https://www.ncbi.nlm.nih.gov/genome/?term=txid50429[orgn]
*Symbiodinium microadriaticum* bulk RNA-seq data (control samples)	Liew et al., 2017	https://www.ncbi.nlm.nih.gov/bioproject/PRJNA205322
*Stylophora pistillata* bulk RNA-seq data	Liew et al., 2018	https://www.ncbi.nlm.nih.gov/bioproject/PRJNA386774
*Stylophora pistillata* bulk RNA-seq data	Rädecker et al., 2021	https://www.ncbi.nlm.nih.gov/bioproject/PRJNA638625

### Resource availability

#### Lead contact

Further information and requests for resources and reagents should be directed to and will be fulfilled by the Lead Contact, Arnau Sebé-Pedrós (arnau.sebe@crg.eu).

#### Materials availability

This study did not generate new unique reagents.

#### Data and code availability

All data are available in GEO repository under accession number GEO: GSE166901. Processed data and annotation tables have been deposited to Mendeley Data: https://doi.org/10.17632/g2mysjfp52.1. Code for reproducing the analysis is available in our lab Github repository (https://github.com/sebepedroslab/Stylophora_single_cell_atlas).

### Experimental model and subject details

#### Adult colony sampling and dissociation

We collected two *Stylophora pistillata* colonies from the Gulf of Eilat/Aqaba. Colonies were sampled from 5 m depth, under a special permit from the Israeli Natural Parks Authority, in front of the Interuniversity Institute of Marine Biology (IUI) in Eilat, Israel. We transferred the adult colonies to the Leon H. Charny School of Marine Sciences at the University of Haifa and maintained them in a closed controlled environment aquarium system, with artificial sea water (ASW, Red Sea Salt, Red Sea Ltd.) and emulating the Gulf of Eilat ambient conditions: salinity of 39, 24°C, and a natural photoperiod.

We dissected adult colonies with a cutter into fragments sized 1–1.5 cm. Fragments were transferred into a small tank with filtered (0.22 μm) ASW (salinity of 39), pre-warmed to 24°C. We repeatedly dissociated colony fragments in 6-well plates in order to ensure a short time between dissociation and cell capture (< 2 h), and cell viability was continuously monitored throughout the sorting process (using Calcein/Draq5 staining, see below). Each fragment was washed with 10 mL of filtered ASW, then washed with 10 mL of Ca-free ASW (CFSW) and transferred into a fresh 10 mL of filtered CFSW with 5 mM EDTA for 5 min. Then each colony fragment was transferred to a new well with a fresh 10 mL of filtered CFSW and 5 mM EDTA and disassociation was performed by gently scraping and peeling the coral tissue from the skeleton with 10 μl sterile plastic tip. The whole dissociation process was performed at room temperature (RT) and took around 10–15 min. We strained the cell suspension twice through a 70 μm cell strainer (PluriStrainer # 43-50070-51), and then collected cells into 15 mL tubes that were placed on ice until FACS-sorting. Cells were stained with 1 μM Calcein violet AM (Invitrogen, #C34858) and 16.6 μM Draq5 (Thermo #62251).

#### Planulae collection and dissociation

We collected larvae from spawning adult *S. pistillata* colonies, under a special permit from the Israeli Natural Parks Authority, in front of the Interuniversity Institute of Marine Biology (IUI) in Eilat, Israel. Traps were made of 160 μm plankton nets attached to a plastic container. We placed traps on 16 adult corals for several nights before the full moon of May 2018. Swimming larvae were kept in 1-l plastic containers for < 24 h before dissociation. To obtain primary polyps, we kept larvae in 1-l plastic containers for 3 days. Metamorphosis and settlement processes proceeded naturally in the plastic containers.

We dissociated 50 specimens for each stage (larvae/polyp), processing 25 specimens at a time to ensure a short time between dissociation and cell capture (< 2 h). Planulae were transferred into 70 μm strainers, washed twice with filtered CFSW and incubated at RT in CFSW with Liberase TM (Roche, #05401119001) at a concentration of 50 mg/ml for 20 min with occasional pipetting. The dissociation reaction was stopped by adding 1/10 volume of 500 mM EDTA and the cell suspension was strained twice through a 70 μm cell strainer (PluriStrainer # 43-50070-51), collected into 15 mL tubes and placed on ice. Cells were stained with 1 μM Calcein violet AM (Invitrogen, #C34858) and 16.6 μM Draq5 (Thermo #62251).

### Methods details

#### Massively Parallel Single-Cell RNA-seq (MARS-seq)

Live single cells were selected using a FACSAria II cell sorter. To this end, we sorted only Calcein positive/Draq5 positive cells, and doublet/multiplet exclusion was performed using FSC-W versus FSC-H (Figure S1). In addition, we employed a specific FACS-sorting strategy to target coral alga-hosting cells containing symbionts: we selected for the strong autofluorescence of the algal symbiont combined with calcein signal, which is specific to coral cells (Figure 4). In all cases, cells were distributed into 384-wells capture plates (all coming from the same plate production batch) containing 2ul of lysis solution: 0.2% Triton and RNase inhibitors plus barcoded poly(T) reverse-transcription (RT) primers for single cell RNA-seq. Fresh cell dissociates were prepared every 2h and sorted plates were immediately spun down at 800 xg, to ensure cell immersion into the lysis solution and then frozen at −80°C until further processing.

Single cell libraries were prepared using MARS-seq (Keren-Shaul et al., 2019). For each stage, all single cell libraries were prepared in parallel: 26,880 libraries for adult colonies (70 plates, including 11 targeted host cell plates), 4,224 for primary polyps (11 plates) and 6,144 for swimming larvae (16 plates) (Table S1). First, using a Bravo automated liquid handling platform (Agilent), mRNA was converted into cDNA with an oligo containing both the unique molecule identifiers (UMIs) and cell barcodes. 0.15% PEG8000 was added to the RT reaction to increase efficiency of cDNA capture. Unused oligonucleotides were removed by Exonuclease I treatment. cDNAs were pooled (each pool representing the original 384-wells of a MARS-seq plate) and linearly amplified using T7 *in vitro* transcription (IVT) and the resulting RNA was fragmented and ligated to an oligo containing the pool barcode and Illumina sequences, using T4 ssDNA:RNA ligase. Finally, RNA was reverse transcribed into DNA and PCR amplified. The size distribution and concentration of the resulting libraries were calculated using a Tapestation (Agilent) and Qubit (Invitrogen). scRNA-seq libraries were pooled at equimolar concentration and sequenced to saturation (≥6 reads/UMI, in most cases > 10 reads/UMI) on an Illumina NextSeq 500 sequencer and using high-output 75 cycles v2.5 kits (Illumina). We obtained 2,210M reads in total, resulting in a median of 34,846 uniquely mapped reads/cell.

#### Paraffin histological sections

*Stylophora pistillata* fragments (1-2cm) were cut from the colony and washed with filtered ASW, then placed in 10ml of filtered ASW. Polyps were relaxed and immobilized by adding MgCl_2_ to final concentration of 3%. After 15min coral fragments were fixed with 4% paraformaldehyde, overnight in 4°c. Decalcification of coral skeleton was carried out using formic acid and sodium citrate until complete immersion of the skeleton (Rinkevich and Loya, 1979). Tissue was rinsed in fresh water and dehydrated through a series of ethanol solutions and butanol and embedded in paraffin. Specimens were sectioned into 8-10μm slices and laid on positively charged slides (Fisherbrand #1255015).

#### *In situ* hybridization

Riboprobes were generated for the specific marker genes (chosen as describe above) by transcription of PCR products, with T7 promoter linked to the 3′ end of the amplicon using the MAXIscript T7 Transcription Kit (ThermoFisher #AM1312) in the presence of digoxigenin labeled UTP (Roche #3359247910). The products of *in vitro* transcription were then treated with DNase. Riboprobes were purified and cleaned using Nucleospin RNA clean-up kit (#740903). The primers used are listed in Table S3. *In situ* hybridization on histological sections was performed. In brief, deparaffinization was done by a series of xylene washes followed by rehydration with a series of ethanol washes. Slides were then permeabilized with 10 μg/mL Proteinase K, for 20 min, at 37°C, washed with PBS treated with Diethyl Pyrocarbonate (Sigma-Aldrich #D5758) and refix with 4% paraformaldehyde for 15 min at RT. Prehybridization and hybridization was performed in humidified hybridization chamber at the desired hybridization temperature (58-60°C) over night. Probe concentration was varied from 5ng/μl to 10ng/μl. After repeated washes with formamide and 1XSSC, specimens were blocked with blocking buffer for 2h at RT and then incubated with anti-Digoxigenin (Roche #11333062910) 1:4000, overnight at 4°c. Slides were washed with AP buffer (100 mM Tris-HCl pH 9.5, 150 mM NaCl, 2 mM Levamisol, 5 mM MgCl_2_) and developed in the presence of BCIP/NBT substrate (Roche #11681451001) at RT in the dark for 2-8h. Reaction was stooped with PTW and background staining was cleared with 70% ethanol. Sections were sequentially cleared with 80% glycerol and imaged with LEICA DM2000 microscope.

### Quantification and statistical analysis

#### MARS-seq gene intervals definition

We used the NCBI *S. pistillata* gene annotation (GCA_002571385.1), but added gene models from a previous annotation (http://spis.reefgenomics.org/) (Voolstra et al., 2015, 2017) not overlapping with NCBI genes. MARS-seq is a 3′-biased RNA-seq method and we observed most of the obtained reads mapping outside annotated *S. pistillata* gene 3′ ends. This is a common problem in non-model species and generally due to limited 3′ UTR annotation (Sebé-Pedrós et al., 2018a, 2018b). To correct this effect and maximize the sensitivity of our scRNA-seq, we extended gene intervals using MARS-seq data as follows:

1. We removed cell barcodes and UMIs from MARS-seq reads using FASTX-Toolkit.
2. We mapped reads to the S. pistillata genome using STAR (Dobin et al., 2013), with parameters:–outFilterMultimapNmax 20–outFilterMismatchNmax 5–alignIntronMax 3500
3. We used the resulting BAM alignment files to define genomic intervals with MARS-seq signal, using MACS2 (Zhang et al., 2008) with parameters: *-g 434000000–keep-dup 20 -q 0.01–shift 1–extsize 20–broad–nomodel–min-length 30*
4. We used the resulting stranded MARS-seq genomic intervals to elongate the 3′ end of *S. pistillata* gene models, allowing a maximum distance of 2Kb between the annotated 3′ end of a gene and the downstream MARS-seq genomic interval (which in addition must be on the same strand and not overlapping with any downstream gene).
5. Finally, to account for potentially unannotated genes, we kept any remaining MARS-seq genomic intervals longer than 100bp. We fused those within 4.5Kb (median gene size in *S. pistillata*) and used these fused intervals as additional gene annotations in our scRNA-seq analysis.

In addition, we systematically looked for any potential *Symbiodinium microadriaticum* contamination in *S. pistillata* assembly, which would confound our host-symbiont transcriptome analysis. To this end, we searched *S. pistillata* scaffolds against NCBI non-redundant nucleotide database, using TBLASTN with parameters *–evalue 1e-5 -max_target_seq 30*. We found only 8 scaffolds with significant (in most cases partial) hits against *Symbiodinium* or other dinoflagellates (NW_019218034, NW_019218831, NW_019219871, NW_019223347, NW_019219627, NW_019219628, NW_019220190, NW_019221271). We manually examined these scaffolds and excluded the potential contaminant genomic regions (and the gene intervals within) flanked by Ns (indicative of potential contamination of a *Symbiodinium* contig assembled within a *S. pistillata* scaffold).

#### MARS-seq reads processing and filtering

To quantify single-cell gene expression, MARS-seq reads were first mapped onto the *S. pistillata* genome using STAR (Dobin et al., 2013) (with parameters: *–outFilterMultimapNmax 20–outFilterMismatchNmax 8–alignIntronMax 3500*) and associated with the modified gene intervals described above. Mapped reads were further processed and filtered as previously described (Keren-Shaul et al., 2019). Briefly, UMI filtering includes two components, one eliminating spurious UMIs resulting from synthesis and sequencing errors, and the other eliminating artifacts involving unlikely IVT product distributions that are likely a consequence of second strand synthesis or IVT errors. The minimum FDR q-value required for filtering in this study was 0.02.

To quantify single-cell gene expression in symbiont cells, we applied the same strategy to MARS-seq reads but this time mapped reads to the *Symbiodinium microadriaticum* genome, the dominant dinoflagellate symbiont species in *S. pistillata* in shallow waters (Aranda et al., 2016; Martinez et al., 2020). For the comparison of *Symbiodinium* inside coral against free-living *Symbiodinium*, we used published transcriptomics data from cultures grown under standard conditions (“control” samples) (Liew et al., 2017). We mapped these RNA-seq reads using the same STAR parameters used for the MARS-seq, then transformed raw counts into CPMs using EdgeR (Robinson et al., 2010) and finally averaged expression between replicates. To join and compare these free-living *Symbiodinium* expression values with those derived from scRNA-seq data, we first aggregated scRNA-seq expression per gene and then quantile normalized the two expression vectors.

#### Metacell analysis

We used Metacell (Baran et al., 2019) to select gene features, construct cell clusters (termed metacells), and to generate data visualizations. We first filtered out cells based on their total UMI counts, excluding cells with low (< 100 UMIs in the adult dataset, < 150 for polyp and < 200 for larva) or very high number of molecules (> 8,000 UMIs). We selected feature genes using normalized size correlation (Baran et al., 2019; Sebé-Pedrós et al., 2018a) threshold of −0.05 and normalized niche score (Baran et al., 2019) threshold of 0.01, additionally filtering for genes with > 1 UMI in at least three cells and a total gene UMI count > 30 molecules (the empirical median marker UMI count was 849 for the adult, 325 for the polyp and 527 for the larva; Figure S1). We excluded from the marker gene lists any ribosomal proteins and histones. In total, we used 809 markers for the adult dataset, 501 for the polyp, and 513 for the larva.

For kNN graph building we used K = 100 target number of edges per cell, and for metacell construction we used K = 30, minimum module size of 20 (10 for both polyp and larva), and 1,000 iterations of bootstrapping with resampling 75% of the cells. This way we obtained an estimate of co-clustering frequency between all pairs of single cells and identified robust clusters of single or grouped metacells (Figure S1). For downstream analyses, we represent gene expression by computing a regularized geometric mean within each metacell and dividing this value by the median across metacells, as implemented in the Metacell package (Baran et al., 2019). We refer to this normalized gene expression values as fold-change (FC) across the manuscript.

After defining an initial metacell set, we filtered out low-quality metacells expressing less than 10 marker genes (25 for polyp and 40 for larva), and with total UMI count below UMI distribution peak, with only three exceptions: we kept metacells regardless of marker gene counts and UMI counts if they (*i*) specifically expressed more than two TF genes (FC > 2), (*ii*) if they had a strong *Symbiodinium* signal (> 10,000 UMIs), or (*iii*) if they expressed any cnidocyte markers (FC > 1.5, in larva dataset where cnidocytes were particularly difficult to capture). Overall, this semi-supervised filtering approach ensures that reported metacells represent highly specific transcriptional states.

For 2D projections (Figures 1D–1F) of adult, polyp and larva datasets, we used different combinations of kNN parameters (K = 30, 30 and 40, respectively) and maximum module graph degrees (degree = 5.5, 4 and 3 respectively). Single cell gene expression heatmaps we show top 10 genes with FC > 2 per metacell (Figure S2).

For downsampling re-analysis (Figures S1G–S1J), we first randomly downsampled reads at the desided target (90% to 5%) using samtools, then recomputed single-cell UMI matrices and finally we performed metacell clustering keeping the same parameters described above. We used this dowsampled matrices to estimate sequencing saturation (Figure S1G) and to assess the stability of our cell type definitions (Figures S1H and S1I), which we estimate as the percentage of overlap of individual cells between our original cell type groupings and the cell type grouping after read downsampling.

For comparison of single-cell RNA-seq versus bulk RNA-seq data, we re-mapped reads from two studies (Liew et al., 2018; Rädecker et al., 2021) using the same STAR parameters used to map scRNA-seq reads and we used STAR gene count estimates with the same gene intervals defined for our scRNA-seq analysis for compatibility in the comparison.

To perform cross-species comparisons, we re-analyzed previously published scRNA-seq datasets for *N. vectensis* (Sebé-Pedrós et al., 2018a), *Xenia* sp. (Hu et al., 2020) and *H. vulgaris* (Siebert et al., 2019) using a similar Metacell-based strategy as the one described above for *S. pistillata*, with only minor modifications of the pre-processing of each dataset. In the case of *N. vectensis*, we used all available MARS-seq data (Sebé-Pedrós et al., 2018a), we re-processed raw reads with same parameters as defined above for *S. pistillata* (e.g., using STAR read mapping instead of bowtie2 as in the original publication), and we filtered out cells with less than 100 or more than 10,000 UMIs. In the case of *Xenia*, we re-processed raw reads using 10x genomics CellRanger software with default parameters, but forcing a high number of reported cells (*–force-cells = 50000*), instead of using the default CellRanger empty droplet call. This aims at ensuring that specific cell types with low UMI counts are not filtered out. In addition, we applied a similar strategy as in *S. pistillata* (see above), to extend 3′ end of *Xenia* sp. gene intervals to match scRNA-seq signal. From the four original datasets (Hu et al., 2020), we discarded one of them (whole organism 10x v2 chemistry) as we detected strong batch effect in that specific experiment. We filtered out cells with less than 300 or more than 10,000 UMIs. Finally, for *H. vulgaris* we used pre-computed UMI matrices, which were already filtered for cells with > 500 and < 50,000 UMIs (Siebert et al., 2019). Parameters for feature genes selection were the same as described previously, except for normalized size correlation threshold of −0.08 used for *Xenia* sp. and *H. vulgaris*, and normalized niche score threshold of 0.05 for *H. vulgaris* and *N. vectensis* datasets. We used K = 100 target number of edges per cell in kNN graph, and K = 30 for metacell construction, with a minimum module size of 20, 20 and 15 for *N. vectensis*, *Xenia* sp. and *H. vulgaris*, respectively, and with 75% cells bootstrapping. We filtered out low-quality metacells as described before, using marker genes thresholds of 30 and 70 for *N. vectensis* and *Xenia* sp. datasets, For *H. vulgaris*, we transferred cluster annotations from the original publication by majority voting and filtered out metacells labeled as biological doublets (Siebert et al., 2019).

#### Cross-stages clustering

For cross-stages comparisons, we grouped metacells into cell types and we computed a regularized gene expression matrix at the cell type level, using the same method described above for metacells. Then, we combined the adult, polyp and larva expression matrices using the union of all genes expressed in the three stages. We performed 1,000 iterations of calculating Pearson’s correlation between cell types based on 75% downsampling of 5,881 variably expressed genes (FC > 1.8) followed by average hierarchical clustering (500 iterations with h = 0.75 and another 500 with h = 0.95). From this, we constructed the co-occurrence matrix for all pairs of cell types (Figure S3A), which we hierarchically clustered to build the cross-stages cell type tree (Figure 2). Finally, we calculated the support for each node in the tree by performing additional 1,000 iterations of clustering with 75% downsampling and reporting the percentage of trees in which each node appears, and we reduced the nodes with less than < 10% support to polytomies.

Next, we searched for genes that are characteristically shared by groups of cell types, which we termed *node-supporting genes*. We selected node-supporting genes for particular groups of cell types as those genes that are expressed in all the descendant tips of that particular node (foreground, FC > 1.7) but not in all the other cell types (background, FC < 1.8). Node-supporting genes selection with different thresholds can be explored in the interactive Shiny (Chang et al., 2020) application (https://sebe-lab.shinyapps.io/Stylophora_cell_atlas/), and it is also possible to define different stringency for node-supporting genes selection by modifying *leakiness* parameters - these specify the percentage of nodes in either foreground or background in which the expression of a gene can be below or above the specified threshold, respectively, for the gene to still be considered a node-supporting gene. Finally, in the interactive application it is also possible to retrieve node-supporting genes that are differentially expressed in selected node relative to all the other descendants of its parent node (termed ‘features versus sister clade’), as well as those that are differentially expressed expressed in all the other tips relative to the tips descending from the selected node (termed ‘features out’).

#### Orthology inference and gene age estimation

In order the infer orthologous pairs between *S. pistillata, N. vectensis, Xenia* sp *and H. vulgaris* we applied the Broccoli algorithm (Derelle et al., 2020) to the predicted proteomes of 35 species, with parameters *-nb_hits 6 -ratio_ortho 0.5* and based on reciprocal DIAMOND (Buchfink et al., 2015) searches with default parameters. Our taxon sampling includes 13 cnidarian genomes (Baumgarten et al., 2015; Bhattacharya et al., 2016; Jeon et al., 2019; Liew et al., 2016; Voolstra et al., 2015, 2017; Ying et al., 2018), 20 non-cnidarian metazoan genomes and 2 unicellular outgroups (Table S2).

We expanded this dataset with additional 25 eukaryotic proteomes in order to estimate *S. pistillata* gene ages/phylostrata (Table S2). We applied Broccoli again to this 60 species dataset and parsed the inferred orthologs using a strict Dollo parsimony criterion in order to generate an age estimation for each *S. pistillata* gene. We applied the same strategy to infer gene ages in *Symbiodinium microadriaticum*, but in this case we used a different taxon sampling of 87 eukaryotic predicted proteomes, including multiple recently sequenced Symbiodiniaceae genomes/transcriptomes (Aranda et al., 2016; Ladner et al., 2012; LaJeunesse et al., 2018; Liew et al., 2016; Parkinson et al., 2016; Voolstra et al., 2015) (Table S2).

#### Gene functional annotation

We generated the following functional annotations for the predicted protein sets of *S. pistillata*, *Xenia* sp., *N. vectensis* and *H. vulgaris*: (*i*) Pfam domain architectures using Pfamscan and the Pfam database (Punta et al., 2012) (version 33.0); (*ii*) Gene Ontologies from the eggNOG database (Huerta-Cepas et al., 2016a) (version 5.0), using eggNOG-mapper (Huerta-Cepas et al., 2017); and (*iii*) gene names from the corresponding best pairwise alignments among predicted proteins from the human genome (version GRCh38, annotations from Ensembl release 100), obtained with the DIAMOND aligner (Buchfink et al., 2015).

We also applied this functional annotation pipeline to the *Symbiodinium microadriaticum* predicted proteome. In this case, however, we used *Arabidopsis thaliana* predicted proteins as the reference species for gene name annotation (version TAIR10, annotations from Ensembl Plants release 47).

Gene ontology enrichment analysis was performed in topGO using classic algorithm (Alexa et al., 2006). Enriched GO terms associated with biological processes (BP), cellular components (CC) and molecular functions (MF) were determined by Fischer test with BH adjustment of p values. All annotated *S. pistillata* genes served as background.

In addition, for a selected list of gene families we refined the annotations using maximum-likelihood phylogenetic analyses. This annotation procedure was applied to transcription factors, myosins, chromatin associated proteins, RNA binding proteins, extracellular matrix proteins and proteins involved in signaling pathways (Table S2). For each gene family, we searched one or more HMM profiles obtained from the Pfam database (version 33, listed in Table S2) against the complete predicted proteomes of the same 35 species used in the genome-wide orthology inference analyses. These searches were performed using the hmmsearch tool in HMMER3 (Mistry et al., 2013), using the GA threshold defined in each Pfam HMM model. Then, we partitioned the non-redundant sets of proteins retrieved for each gene family into homology groups of similar sequences, using all-to-all pairwise protein alignments (DIAMOND aligner (Buchfink et al., 2015) in *sensitive* mode; and a highly-inclusive clustering step (based on MCL (Enright et al., 2002) using *abc* mode and a low inflation parameter = 1.15). We built multi-sequence alignments of each homology group with MAFFT (Katoh and Standley, 2013) (using up to 10,000 rounds of iterative refinement and the E-INS-i algorithm; trimmed the alignments using ClipKIT (Steenwyk et al., 2020) (retaining parsimony-informative and constant sites and removing sites with a gap threshold over 0.7). The trimmed alignments were used to obtain gene trees with IQ-TREE (Nguyen et al., 2015), using up to 10,000 refinement iterations and a convergence threshold of 0.99. The best-fitting evolutionary model was chosen independently for each gene tree, based on the BIC criterion. Phylogenetic statistical supports were calculated using the UF bootstrap procedure (1000 replicates) (Hoang et al., 2018). Finally, we retrieved pairs of orthologs and orthology groups from each gene tree using the species overlap algorithm implemented in the ETE3 Python library (Huerta-Cepas et al., 2016b).

#### Cross-species clustering

For cross-species comparisons, in each dataset we first grouped metacells in cell types and we computed a regularized gene expression matrix at the cell type level, using the same method described above for metacells. To compare *S. pistillata* cell types with those of the other species, we generated pairwise datasets of orthologous gene expression, allowing here for one-to-many orthology relationships by duplicating genes entries in one species when it had up to three expressed orthologs in another species. This resulted in 18,478 *S. pistillata*-*N. vectensis* pairs, 15,917 *S. pistillata*-*Xenia* sp. And 12,900 *S. pistillata*-*H. vulgaris* pairs. The resulting joined cell type expression matrices were first quantile normalized and then we calculated Kullback–Leibler divergence (KLD) for all cell type pairs. Links between individual cell types pairs with lowest KLD (0.98 quantile) are shown in circos diagrams (Figure 3B), generated using the R package circlize (Gu et al., 2014). We selected individual neuronal and gland cell types to compare between species as follow: *S. pistillata* cell types that had top links to only one cell type in any of the other species, or cell types that were functionally annotated (three neuronal cell types in *Nematostella*), had top link to a single cell type in *Stylophora*, and shared at least 10 variably expressed orthologs (FC > 1.3) with other species. For resulting groups of cell types we compared the expression of shared orthologs (FC > 1.3) to their expression in other cell types; significance was calculated using paired Wilcoxon test between in- and outgroup cell types. We also compared host and gastrodermal cells between *S. pistillata* and *Xenia* sp. using 1,929 variable orthologs (FC > 1.5) expressed in these cell types (Figure 4G).

To compare all four species, we further grouped cell types in each species dataset into broad cell types, and combined expression data for 4,523 one-to-one orthologs expressed in all four species. This joint cross-species matrix was quantile normalized. We then performed clustering and broad cell type tree generation using 1,227 variable genes (FC > 1.8), as described before for cross-stages dataset. The resulting tree (Figure 2) and node-supporting genes (foreground FC > 1.3, background FC < 1.8; see cross-stages section for details) can also be explored interactively in the Shiny application: https://sebe-lab.shinyapps.io/Stylophora_cell_atlas/.

#### Statistical analysis

Statistical analyses were performed using R software (version 3.5). All statistical tests used were two-sided (where applicable) and are indicated in the figure legends. The “n” for each analysis is indicated in all relevant figure panels and corresponds to the number of genes involved in each analysis. No specific methods were used to determine whether data met assumptions of the statistical approach.

### Additional resources

The dataset can be interactively explored (and data downloaded) in https://sebe-lab.shinyapps.io/Stylophora_cell_atlas/. We show there detailed 2D projections and gene expression maps for all three life stages of *Stylophora pistillata*. Additional interactive functionalities include inspecting the expression of individual genes or groups of genes, and retrieving specifically expressed genes in metacells or cell types, using user-defined thresholds. We also provide interactive interfaces for exploring *Stylophora pistillata* cell type tree and broad cell type tree for the four species analyzed in this manuscript, where it is possible to retrieve genes supporting cell type similarities at different levels in the trees, and using different filtering thresholds. Finally, pairwise species cell types similarities can be explored using different distance metrics and varying sets of orthologs, and it is also possible to inspect genes supporting each cell type similarity.

## Supplemental information

Table S1. *S. pistillata* scRNA sequencing library statistics, related to Figure 1

Table S2. Gene orthology and annotation, related to Figures 1, 2, and 3 Taxon sampling lists employed for orthology inference and phylostratigraphic analyses. Also includes information on Pfam domains employed for phylogeny-based annotations (see STAR Methods).

Table S3. Primers for *in situ* hybridization probes, related to Figures 1, 4, and 5
